# Hemoglobin Derivatives in Beef Irradiated with Accelerated Electrons

**DOI:** 10.3390/molecules28155773

**Published:** 2023-07-31

**Authors:** Ulyana Bliznyuk, Polina Borshchegovskaya, Alexander Chernyaev, Victoria Ipatova, Aleksandr Kozlov, Oleg Khmelevskiy, Irina Mezhetova, Alexander Nikitchenko, Igor Rodin, Elena Kozlova

**Affiliations:** 1 Department of Physics, Lomonosov Moscow State University, GSP-1, 1-2 Leninskiye Gory, 119991 Moscow, Russianikitchenko.ad15@physics.msu.ru (A.N.); waterlake@mail.ru (E.K.); 2 Skobeltsyn Institute of Nuclear Physics, Lomonosov Moscow State University, GSP-1, 1-2 Leninskiye Gory, 119991 Moscow, Russia; ipatova.vs15@physics.msu.ru; 3 Department of Medical and Biological Physics, Sechenov First Moscow State Medical University, 119991 Moscow, Russia; 4 Department of Chemistry, Lomonosov Moscow State University, GSP-1, 1-2 Leninskiye Gory, 119991 Moscow, Russia; 5 Department of Epidemiology and Evidence-Based Medicin, Sechenov First Moscow State Medical University, 119991 Moscow, Russia

**Keywords:** beef, electron beam processing, spectrophotometry, methemoglobin, mathematical modeling, computer simulation

## Abstract

The efficiency of food irradiation depends on the accuracy of the irradiation dose range that is sufficient for inhibiting microbiological growth without causing an irreversible change to the physical and chemical properties of foods. This study suggests that the concentration of hemoglobin derivatives can be used as a criterion for establishing the limit for chilled beef irradiation at which irradiation-induced oxidation becomes irreversible. The express spectrophotometry method for estimating the hemoglobin derivative concentration shows a nonlinear increase in methemoglobin concentration from 15% to 50% in beef irradiated by accelerated electrons with the doses ranging from 250 Gy to 10,000 Gy. The monitoring of the hemoglobin derivative concentration for three days after irradiation shows nonmonotonous dependencies of methemoglobin concentration in beef in the storage time since the oxidation of hemoglobin occur as a result of irradiation and biochemical processes in beef during storage. The proposed method based on the quantitative analysis of the hemoglobin derivative concentration can be used to estimate the oxidation level for irradiation of foods containing red blood cells. The study proposes a model that describes the change in hemoglobin derivative concentration in beef after irradiation considering that oxidation of hemoglobin can be triggered by the direct ionization caused by accelerated electrons, biochemical processes as a result of bacterial activity, and reactive oxygen species appearing during irradiation and storage. This research throws light on the mechanisms behind food irradiation during storage that should be taken into account for selecting the optimal parameters of irradiation.

## 1. Introduction

At present, large food market players in East and South Asia, South America, and Russia give preference to food irradiation treatment in order to increase the shelf life of products and ensure their microbiological safety, which is especially relevant because of the disruption and complication of the logistic chains in post-COVID times [1]. According to ISO 14470:2011 [2], gamma radiation emitted by isotopes ^60^Co and ^137^Cs, electron beams with the energy up to 10 MeV, and bremsstrahlung radiation with the energy up to 5 MeV are used in the food industry, since these values allow the achievement of the desired result without causing induced activity in irradiated objects. Considering that electron beams have proved to be more efficient than gamma radiation generated by radioisotopes, irradiation facilities are frequently equipped with electron accelerators, as they can ensure a higher treatment rate and a diverse penetration depth by varying the energy of electrons [3]. Various beam penetration depths enabled by electron accelerators allow the solving of a wide range of tasks, from an increase in the plant growth rate with doses of 5–20 Gy [4] to sterilization of food with a dose up to 10 kGy [5], which is the maximum permissible dose for food irradiation pursuant to international standards [6]. Despite the fact that the International Organization for Standardization specifies the maximum doses applicable to different categories of food, each type of food should be irradiated within a specific dose range to ensure microbiological safety without detriment to the properties of the final product.

Being highly susceptible to bacterial contamination, meat requires a special approach for selecting the effective irradiation dose range, since undesired changes that may occur in meat upon irradiation depend on a great number of variables because of the presence of different fats, proteins, and carbohydrates in its composition. Moreover, it has been proved that the effect of irradiation depends on the type of meat, pH of tissues, storage temperature, oxygen concentration, type of package, antioxidants present in meat, and other factors [7].

To delineate the scope of physical and chemical changes in irradiated meat, the researchers differentiate between mechanisms behind the change in color, taste, and odor. The discoloration of irradiated meat is known to occur because of the oxidation of pigments and oxy-derivatives of myoglobin [8]. Beef is most susceptible to irradiation-induced color changes compared with other types of meat [9] because of a high myoglobin content, which is extremely sensitive to different chemical changes in the tissues and to the absorption of energy [7,10]. The taste of meat is determined by the composition of water-soluble compounds, which are more abundant in beef than in pork or poultry meat [11]. The intensity of odors emitted by volatile organic compounds, appearing after irradiation, depends on the presence of fat and proteins in meat, as well as on the irradiation dose. Volatile compounds are formed in meat because of protein and lipid oxidation, and their concentration is determined by two competing processes—the decomposition of compounds because of irradiation and accumulation of compounds as a result of the decomposition of other low-molecular-weight compounds and biomacromolecules [12].

Oxidation, microbial growth, and enzymatic autolysis [13] are accountable for the physical and chemical changes occurring during storage, which inevitably alter the structure and organoleptic properties of meat, making it unsuitable for consumption. The formation of reactive oxygen species that attack fatty acids, along with the autoxidation of lipids, are a cause of meat deterioration and the appearance of volatile organic compounds that are responsible for the foul smell associated with meat spoilage [14]. Ferrous ions contained in myoglobin and hemoglobin contribute to the formation of lipid-free radicals that trigger a chain reaction in lipid oxidation [13]. It has been found [15] that mesophilic and psychrotrophic bacteria strains cause various volatile organic compounds to appear because of a big variety of mechanisms, such as the proteolytic activity of strains, lypolysis [16], chemical esterification of alcohols and carboxylic acids contained in meat [17], hydrolysis of triglycerides, phospholipids, and amino acid degradation [18], among other processes. While the formation of volatile compounds in meat immediately after slaughtering that has a bacterial content up to 10^2^ CFU/g is negligibly low, with the passage of time the sigmoidal growth of bacteria populations [19,20] increases the total concentration of volatile compounds [21,22] as a result of intensive chemical changes in meat during storage. The enzymes can combine with organic compounds and trigger chemical reactions, causing the formation of volatile compounds and the breakdown of biomacromolecules [23]. Therefore, it can be concluded that the complexity of meat treatment and storage calls for a search for the generic biochemical markers indicating changes in meat, which would be applicable to a wide range of categories.

It is known that hemoglobin derivatives in red blood cells are sensitive to physical and chemical factors, such as irradiation and the presence of toxins in blood [24], oxygen content [25], and other factors both in vivo and in vitro [26].

In this work, we use express spectrophotometry analysis to study hemoglobin derivatives in beef samples during three days of storage after exposure to accelerated electrons with different doses. Considering that oxidation in irradiated meat during storage manifests in an increase in methemoglobin concentration, the methemoglobin rate can serve as a marker of the biophysical and biochemical processes occurring in beef during storage. In view of the need to find the effective irradiation dose range for such a complex product as beef, we propose a kinetic model that describes the change in concentrations of hemoglobin derivatives in irradiated organic objects containing red blood cells, bearing in mind that hemoglobin oxidation is triggered not only directly by accelerated electrons during irradiation, but also by biochemical processes as a result of microbial growth and reactive oxygen species appearing during irradiation and storage. The study explores the mechanisms behind food irradiation, since it is important to find a balance between reducing the bacterial content to extend the shelf life of meat and meat oxidation so as to not destroy proteins, muscle fibers, and lipids, which are responsible for maintaining the marketable qualities of food including color, odor, and taste.

## 2. Results

### 2.1. Research Stages

**Figure 1:**
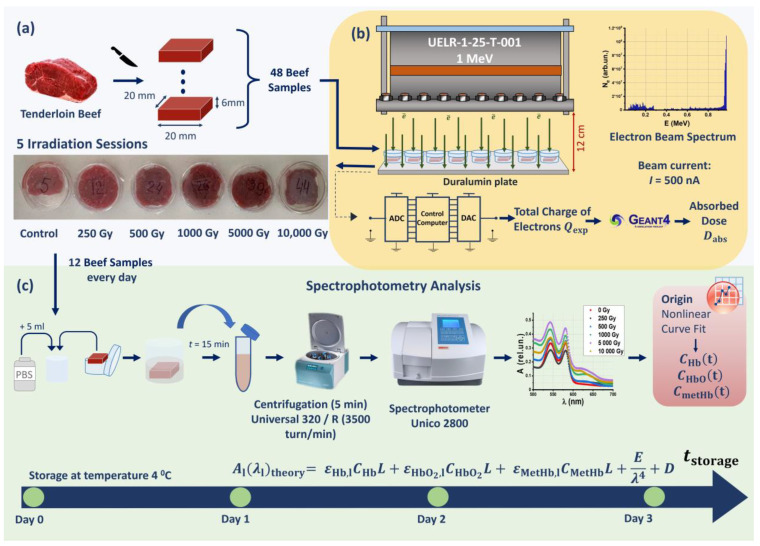
Stages of experimental research: (**a**) preparation of beef samples; (**b**) beef irradiation with accelerated electrons; (**c**) spectrophotometry for hemoglobin derivative analysis.

Beef tenderloin samples were irradiated with 250 Gy, 500 Gy, 1000 Gy, 5000 Gy, and 10,000 Gy using a 1 MeV electron beam accelerator UELR-1-25-T-001. An hour after the exposure, 10 out of 40 beef pieces irradiated with different doses, as well as 2 out of 8 nonirradiated beef pieces, were placed in a buffer solution for measuring the absorption spectra of the hemoglobin derivatives in the beef suspensions. The remaining 30 irradiated samples and 6 nonirradiated samples were subjected to spectrophotometry analysis within the next 3 days after exposure. The three stages of the experiment are shown in Figure 1; details are provided in Materials and Methods.

### 2.2. Nonuniformity of Depth–Dose Distribution across Beef Samples

**Figure 2:**
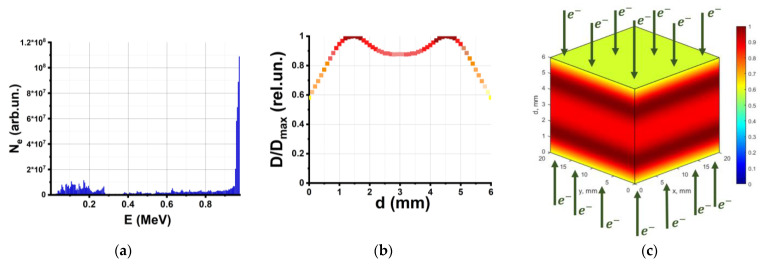
Simulated depth–dose distribution created by 1 MeV electron beam: (**a**) electron beam spectrum; (**b**) depth distribution of the dose absorbed by water parallelepiped layers; (**c**) 3D color map of relative absorbed dose distribution in the water parallelepiped.

To assess the dose absorbed by the samples D_exp_, as well as the dose uniformity, the pieces of beef were represented by 20 mm × 20 mm × 6 mm water parallelepipeds in a computer simulation based on the Geant4 toolkit, which factored in the UELR-1-25-T-001 energy spectrum (Figure 2a) and the irradiation method used for treating beef samples (Figure 1b). The simulation details are outlined in Materials and Methods.

Figure 2b shows the depth distribution of the dose normalized to the maximum dose in the water parallelepiped, which simulates the beef sample during bilateral irradiation. The irradiation uniformity coefficient (K) for the water parallelepiped is calculated according to the formula:

(1) $$K=\frac{D_{\min}}{D_{\max}},\;$$

where D_min_ and D_max_ are the minimum and maximum values, respectively, of the absorbed dose in the water parallelepiped. The calculation shows that the dose uniformity in the beef sample is not more than K = 57 ± 1%. The 3D color map representing the relative absorbed dose distribution throughout the water parallelepiped in Figure 2c is provided to illustrate the dose distribution in the irradiated object. As can be seen, during bilateral irradiation, the maximum dose of 100% is absorbed by the layers at the depth between 1 mm and 2 mm from the surface of the parallelepiped. The relatively low dose uniformity caused by electron beams should be taken into account in the search for optimal food parameters [27]. 

### 2.3. Concentrations of Hemoglobin Derivatives in Beef Samples after Exposure to Accelerated Electrons

One hour after irradiation, spectrophotometry analysis was performed on two nonirradiated samples and on ten beef samples irradiated with different doses to determine the absorption spectra of hemoglobin derivatives in beef suspensions. The concentrations of hemoglobin derivatives in the remaining samples, stored in the fridge at 4 °C, were measured every day during three days after irradiation (Figure 1c).

#### 2.3.1. Theoretical Absorption Optical Spectra and Estimation of Hemoglobin Derivative Concentrations in Beef Suspensions

**Figure 3:**
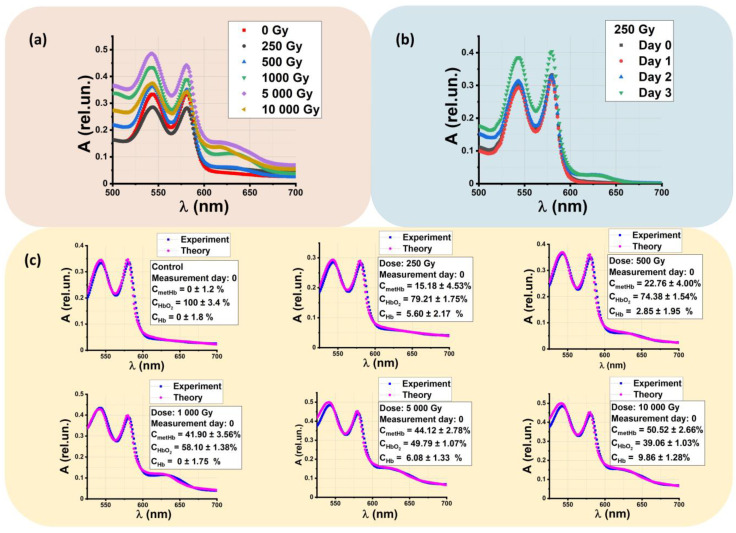
Absorption spectra of beef suspensions A(λ): (**a**) A(λ) measured one hour after irradiation; (**b**) A(λ) obtained from supernatant of beef samples irradiated with 250 Gy measured every day within 3 days after irradiation; (**c**) fitting function $A_{l}{\left(\lambda_{l}\right)}_{\mathrm{theory}}$ calculated based on the experimentally measured A(λ): blue dots are experimentally measured A(λ); pink dots are theoretically calculated, $A_{l}{\left(\lambda_{l}\right)}_{\mathrm{theory}}$. The values indicated on each graph are relative concentrations of hemoglobin derivatives calculated using the nonlinear curve fitting method described in Materials and Methods.

The concentration of hemoglobin derivatives is calculated considering the absorption spectra of beef suspensions made from nonirradiated beef samples and the samples irradiated with 250 Gy, 500 Gy, 1000 Gy, 5000 Gy, and 10,000 Gy. Figure 3a shows the dependencies of the optical density A of beef suspensions, measured one hour after irradiation, on wavelength λ. With an increase in the irradiation dose, the amplitude of the methemoglobin (metHb) at wavelength λ = 628–632 nm slightly increases up to 0.15 rel. un., while the suspension obtained from nonirradiated samples does not show such an increase in optical density at wavelength λ = 628–632 nm. When methemoglobin occurs in beef samples, the whole absorption spectrum of the beef solution changes within 500–700 nm in the irradiated samples. When methemoglobin peaks at 628–632 nm, the amplitudes of oxyhemoglobin peaks of 540 nm and 580 nm decrease with an increase in the irradiation dose.

This phenomenon could occur because of the oxidation of oxyhemoglobin (HbO_2_) to metHb in beef samples after irradiation with accelerated electrons. Figure 3b shows the absorption spectra of beef suspensions made from the beef samples irradiated with 250 Gy measured every day during three days after irradiation. It can be seen that, as time passes, the amplitude of metHb slightly increases under the influence of various processes that occur in beef during storage.

Figure 3c compares the concentrations of hemoglobin derivatives oxyhemoglobin (HbO_2_), methemoglobin (metHb), and deoxyhemoglobin (Hb) measured one hour after irradiation. It can be seen that the metHb concentration in beef suspensions increases when the irradiation dose applied to the beef samples ranges from 250 Gy to 10,000 Gy. The small values of the approximation errors prove the validity of the method used to determine the concentration of the hemoglobin derivatives. To quantify the concentrations of all hemoglobin derivatives, instead of analyzing specific wavelengths, we use the molar absorption spectra of metHb, HbO_2_, and Hb at all wavelengths in the entire range of 500 to 700 nm with a step of 1 nm, since this approach ensures the higher accuracy of estimating hemoglobin derivative concentration.

#### 2.3.2. Dependencies of the Hemoglobin Derivative Concentration in Beef on the Irradiation Dose and Storage Time

**Figure 4:**
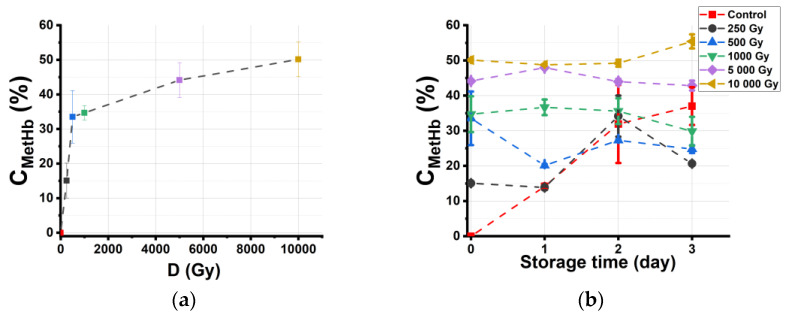
The concentration C_metHb_ in suspensions obtained from beef samples: (**a**) dependency of C_metHb_ on the irradiation dose (D); (**b**) dependency of C_metHb_ in beef samples on storage time t_storage_.

Since oxidation processes caused by ionization radiation, bacterial activity, oxygen, and reactive oxygen species (ROS) occurring as a result of ionizing radiation affect the organoleptic properties and chemical composition of irradiated beef, it is important to monitor the concentration of methemoglobin C_metHb_ during storage. Figure 4a shows the dependency of C_metHb_ in beef suspensions on the dose, which is measured one hour after irradiation. It can be seen that C_metHb_ in irradiated beef increases exponentially with the increase in the irradiation dose from 250 Gy to 5000 Gy, and after the methemoglobin level reaches 50% at the dose of 5000 Gy, it does not show any further increase in methemoglobin level with an increase in the dose of up to 10,000 Gy.

Figure 4b shows the dependencies of C_metHb_ in beef suspensions on the storage time t_storage_. It is evident from the graph that C_metHb_ in nonirradiated samples, used as controls, steadily increases from 0% to 35% during 3 days of observation, and C_metHb_ in the suspension obtained from beef samples irradiated with 250 Gy shows a similar dependency from day 1 and day 2 of storage, when it reaches the peak of 34% to drop to 20% on day 3. While C_metHb_ in the suspensions obtained from beef samples irradiated with 1000 Gy, 5000 Gy, and 10,000 Gy does not change significantly during 3 days of storage, C_metHb_ in the suspension obtained from beef samples irradiated with 500 Gy drops on day 1 to slightly peak on day 2. 

After 3 days of storage, the concentration of oxyhemoglobin C_HbO_2__ in the suspensions obtained from control samples decreases from 100% to 65%, while C_HbO_2__ in the suspensions obtained from beef samples irradiated with a maximum dose of 10,000 Gy decreases from 50% to 45%, which shows that HbO_2_ is present in all the samples during 3 days of storage. The concentration of Hb fluctuates slightly, without a significant change in its value, which never exceeds 10%.

#### 2.3.3. Effective Dose Range for Beef Irradiation

For each food category, there is an effective dose range that reduces bacterial content without causing irreversible physical and chemical changes in the product. There are physical and biological factors that determine the effectiveness of food irradiation. The main physical factor is the irradiation dose uniformity in the irradiated product, since the higher the dose uniformity, the more uniform suppression of microorganisms can be achieved over the volume of the product. Due to the physical nature of electron beam dose distribution, it is impossible to achieve 100% dose uniformity throughout the irradiated product, so there is an established dose range for each category of product, which should be complied with during irradiation.

In the absence of express methods to track chemical changes occurring in animal products after irradiation, we came up with a spectrophotometry method that allows us to estimate the oxidation level in biological objects containing red blood cells by measuring the absorption spectra of hemoglobin derivatives. The proposed method involves the estimation of the oxidation level by determining the methemoglobin concentration in the solutions made from the irradiated beef samples. Using the absorption spectra of beef solutions containing red blood cells, the methemoglobin concentrations in meat were obtained using the specific molar absorption coefficient spectra of hemoglobin derivatives and the nonlinear curve fitting of the spectra. There is a direct relationship between methemoglobin concentration and the oxidation level in beef containing red blood cells, since the color of beef changes from bright red to dark brown after exposure to accelerated electrons because of the oxidation of oxyhemoglobin to methemoglobin as a result of both direct ionization and radicals.

This method proves that the concentration of methemoglobin in beef samples increases from 15% to 50% one hour after irradiation, with the dose ranging from 250 Gy to 10,000 Gy. However, the observation of irradiated samples conducted during the three days after irradiation reveals that the dependency of c in the irradiated beef samples on the dose is nonmonotonous. At the same time, the rate of metHb in the nonirradiated beef samples steadily increases from 0% to 35% during storage. 

Three days after irradiation, the C_metHb_ in the beef samples irradiated with relatively low doses, 250 Gy, 500 Gy, and 1000 Gy, is smaller compared with that in the nonirradiated samples, while C_metHb_ in the samples irradiated with 5000 Gy and 10,000 Gy is greater when compared with that in nonirradiated samples. The fact that the dose-effect dependency has an inflection point at approximately 1000 Gy is critical because irradiation with doses of up to 1000 Gy significantly reduces bacterial content [28] without causing an irreversible transformation of hemoglobin derivatives. Therefore, it can be assumed that beef irradiation with doses ranging from 250 Gy to 1000 Gy is effective for extending the shelf life of beef since, on the one hand, this dose range inhibits bacterial activity, and, on the other hand, it impedes meat oxidation during the three days after exposure. 

In view of our findings, the optimal irradiation parameters for food preservation are selected to ensure that bacteria are suppressed without causing irreversible oxidation of hemoglobin in irradiated beef. Experimental results obtained in the study revealed nonlinear effects of hemoglobin oxidation in irradiated beef over time, which should be taken into account when selecting the irradiation method and effective dose range. It was observed that irradiation with doses up to 1000 Gy does not inactivate all bacteria in the beef samples, which leads to microbial growth in the product; however, this growth is lower than in nonirradiated beef samples, while there is no irreversible oxidation of hemoglobin in the product after exposure to accelerated electrons. Above 1000 Gy, irradiation sufficiently reduces the bacterial content, but the irradiation causes severe oxidation of hemoglobin, which leads to irreversible color change in meat. These results can be taken into account when choosing the optimal dose range of beef irradiation. During the treatment with accelerated electrons, there is irradiation nonuniformity in irradiated beef samples, which should also be taken into account when selecting the optimal irradiation method.

## 3. Discussion

### 3.1. Reasons for Methemoglobin Concentration Change in Beef Samples during Storage after Electron Beam Irradiation

The mechanisms of the transformation of oxyhemoglobin into methemoglobin in beef samples are determined by the irradiation dose absorbed by the beef pieces and the biochemical changes occurring during storage. This chapter will discuss the reasons for the change in metHb concentration in nonirradiated and irradiated beef samples during storage.

#### 3.1.1. Direct Ionization of Fe^2+^ Caused by Accelerated Electrons

The first reason for the increase in metHb concentration in the beef samples is the direct action of accelerated electrons on Fe^2+^ ions. The experimental data in Figure 4a prove that irradiation increases *C*_metHb_ in beef samples one hour after exposure. This observation is consistent with the results of experiments [29,30,31,32] to determine the influence of irradiation on red blood cells (RBCs), which prove that direct ionization triggers the transformation of HbO_2_ to metHb in RBCs.

#### 3.1.2. Bacterial Activity

The second reason for the formation of metHb in the beef samples is the biochemical and biophysical processes caused by bacterial activity. As shown in papers [33,34], the concentrations of microorganisms in meat and poultry, as well as the bacterial content in nutrient solutions [35], increase sigmoidally as the storage time passes; moreover, the rate of bacterial growth depends on a number of factors, such as the initial bacterial content, number and types of bacteria populations, storage temperature, and nutrients [36]. In addition, the sigmoidal character of the dependency of bacterial content on the storage time can also be observed after irradiation, and the rate of bacterial growth decreases with an increase in the dose applied [33]. Paper [37], studying the effect of gamma irradiation emitted by ^60^Co on the hemoglobin content and oxygen consumption of freshwater fish, illustrates that by-products caused by bacterial oxidation of ammonia, such as nitrites and nitrates, trigger the oxidation of HbO_2_ to metHb during storage. Review [13], discussing the causes of the discoloration of meat during storage, points out that bacteria oxidize myoglobin protein, whose amino acid composition is similar to that of hemoglobin. Paper [38] discusses the formation of methemoglobin in vivo as a result of hydrogen peroxide, H_2_O_2_, formed by bacteria. Proteolytic enzymes produced by anaerobic pathogenic bacteria *Prevotella intermedia* and the antibiotic pyocyanin produced by bacteria *Pseudomonas aeruginosa* also cause the formation of methemoglobin in the human body. In summary, it is clear that bacterial activity causes methemoglobin to form both in vivo and in vitro.

Based on the sigmoidal growth of bacteria and our experimental results shown in Figure 4b, it can be suggested that the dependency of *C*_metHb_ in the nonirradiated samples on the storage time has a sigmoidal character during the observation period of 3 days.

#### 3.1.3. Reactive Oxygen Species

The third reason for the increase in metHb concentration in the beef samples is the autoxidation caused by oxygen coming into contact with beef during storage. Considering that at least 3% of the hemoglobin molecules undergo autoxidation every 24 h because of oxidants present in blood [39], the amount of metHb, which is formed in the beef samples in the presence of oxygen during storage, depends on a variety of factors, such as oxygen concentration and storage temperature. When meat is stored at the temperature of 2–5 °C, molecular oxygen, acting as an oxidant, forms ROS, such as H_2_O_2_, HO^•^_2_, O_2_^•–^, and HO^•^. According to the Fenton reaction, Fe^2+^ converts into Fe^3+^ in the presence of hydrogen peroxide, H_2_O_2_, which leads to an increase in methemoglobin concentration. In this reaction, the H_2_O_2_ molecules are converted into HO^•^ free radicals that can initiate the lipid peroxidation forming organic radicals that also can oxidize hemoglobin [40,41]. 

ROS caused by the indirect action of accelerated electrons, which appear as a result of radiolysis, form metHb in organic objects, and the higher the dose, the greater the metHb concentration that can be observed. Moreover, a high concentration of water in food prompts the formation of a greater amount of ROS, which causes a variety of chemical changes in organic objects [42]. This fact should be taken into account for selecting the irradiation doses applicable to the products with high water content.

#### 3.1.4. Autoreduction of Methemoglobin to Oxyhemoglobin

The reason for the decrease in metHb concentration in the beef samples during storage is that it can be caused by the autoreduction of metHb to HbO_2_, which occurs because of different antioxidant processes. Figure 4b proves that metHb concentration in the beef samples irradiated with doses of 250 Gy and 500 Gy decreases one day after irradiation, which provides the evidence that autoreduction occurs in beef samples during storage.

Recent studies suggest that metHb can transform into HbO_2_ in vivo because of the action of antioxidants [43], such as glutathione molecules, vitamins C and E, and the enzyme methemoglobin reductase [39,41,44]. Adding perfluorocarbon compounds, antioxidants such as cytoflavin, to the blood in vitro reduces metHb concentration, which otherwise increases over time [39,41]. Therefore, both in vivo and in vitro metHb can transform into HbO_2_ as a result of autoreduction, which should be taken into account in the treatment of products that contain RBCs.

Summarizing all the processes occurring in the beef samples after irradiation, it can be concluded that the metHb level is determined by oxidative processes because of the direct action of accelerated electrons, bacterial activity, and autoxidation of hemoglobin caused by ROS, as well as by the autoreduction of metHb during storage. The methemoglobin level indicates, on the one hand, how many bacteria are inactivated by the irradiation, and on the other hand, it shows the degree of meat oxidation caused by the radiation. The dependency of the methemoglobin concentration on time shows whether bacterial processes are developing actively during storage. If the level of methemoglobin increases with the storage time, it means that we have not reached the lower limit of the optimal dose range for beef irradiation. The dependency of methemoglobin concentration on the irradiation dose shows how much the meat has been oxidized by irradiation. If the hemoglobin oxidation is irreversible and no autoreduction of methemoglobin to oxyhemoglobin is observed, it means that we have exceeded the upper limit of the dose range. Therefore, the methemoglobin rate in beef during storage serves as an indicator of whether the irradiation dose range has been met.

### 3.2. Kinetics of Methemoglobin Concentration in Beef Samples after Irradiation

**Figure 5:**
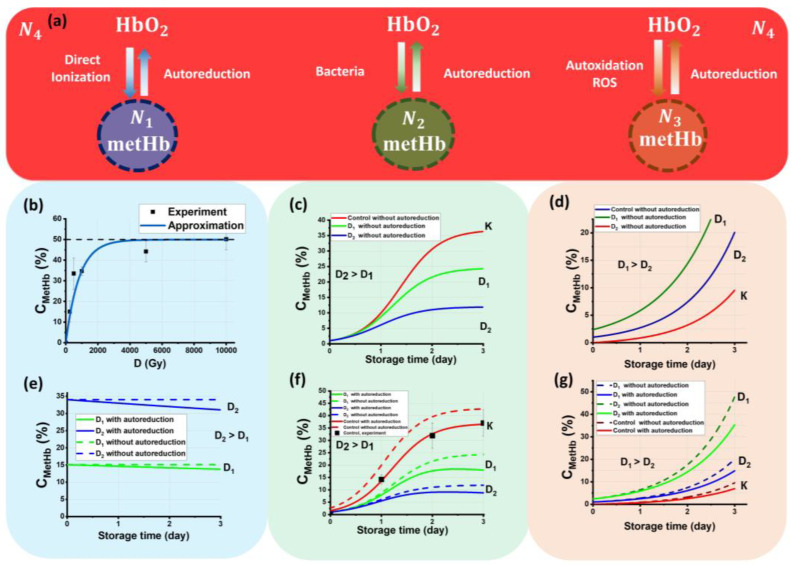
The processes occurring in beef samples during storage after irradiation: (**a**) transformations of metHb and HbO_2_; (**b**) the dependency of *C*_metHb_ on the dose because of the direct ionization of Fe^2+^ caused by accelerated electrons; (**c**) the dependency of *C*_metHb_ on the storage time because of bacterial activity; (**d**) the dependency of *C*_metHb_ on the storage because of autoxidation caused by ROS; (**e**) the dependency of *C*_metHb_ on the dose because of direct ionization of Fe^2+^ with concurrently occurring autoreduction of metHb to HbO_2_; (**f**) the dependency of *C*_metHb_ on the storage time because of bacterial activity with concurrently occurring autoreduction of metHb to HbO_2_; (**g**) the dependency of *C*_metHb_ on the storage because of autoxidation with concurrently occurring autoreduction of metHb to HbO_2_.

To increase the effectiveness of beef irradiation, it is important to determine the dose range that would reduce the bacterial content in meat to extend its shelf life without altering the organoleptic properties and appearance of the product. Setting the criteria for choosing upper and lower limits of the irradiation dose range becomes easier based on a model that describes the microbiological and chemical parameters of meat after exposure to accelerated electrons. The model represented in Figure 5 assumes that hemoglobin molecules in the beef samples irradiated with electrons are dynamic statistical ensembles impacting each other throughout the entire period of storage.

Figure 5a represents the following ensembles:

- Ensemble *N*_1_ is metHb molecules that appear because of the transformation of ions Fe^2+^ into Fe^3+^ as a result of the direct action of accelerated electrons during irradiation;
- Ensemble *N*_2_ is metHb molecules that are formed as a result of bacterial activity during the entire period of storage;
- Ensemble *N*_3_ is metHb molecules that are formed because of the autoxidation of HbO_2_ in the presence of ROS present both in nonirradiated and irradiated samples;
- Ensemble *N*_4_ is a pool of HbO_2_ molecules that can transform into metHb molecules because of oxidation and back into HbO_2_ molecules because of autoreduction during the observation time.

The main assumptions of the model in Figure 5a are:

- Each ensemble occurs because of specific factors, such as exposure to the direct action of accelerated electrons, bacterial activity, and autoxidation.
- At the beginning of the observation, t = 0 hemoglobin molecules *N* in the beef samples are represented only as HbO_2_ molecules, i.e., *N*(t = 0) = *N*_4_(t = 0) = 100%. As further dynamic transformation of hemoglobin derivatives occurs, the sum of all molecules in four ensembles is always equal to 100%.
- The model assumes that mutual transformations of HbO_2_ into metHb and back occur during the observation time, and the process of HbO_2_ transition into deoxyhemoglobin Hb and back is not taken into account. Hb concentration is assumed to be 0% during the whole observation time.

Since hemoglobin molecules are equally distributed in the whole volume of each beef sample, and the concentration of hemoglobin is proportional to the number of hemoglobin molecules, all dependences describing the kinetics of ensembles are valid for the concentrations of hemoglobin derivatives.

#### 3.2.1. The Kinetics of Ensemble N_1_

The ensemble *N*_1_ is determined by the direct action of accelerated electrons, so it is determined by the physical properties of irradiation, such as the effective cross-section σ of the interaction of electrons with an Fe^2+^ ion, which corresponds to the size of the Fe^2+^ ion of approximately 10^−16^ cm^2^ and the electron flux density *φ_N_* calculated by the following formula:

(2) $$\varphi_{N}=\frac{\Delta N_{e}}{\Delta S\Delta t},\;$$

where ∆*N_e_* is the number of electrons intersecting the surface area ∆*S* perpendicular to the initial direction of the electrons in the beam during the irradiation time ∆*t*. According to beef irradiation data obtained during the experiment and calculations discussed in Materials and Methods, the average electron flux density is equal to 3 × 10^10^ cm^−2^ s^−1^.

It is assumed that the number of Fe^2+^ ions $N_{{\mathrm{Fe}}^{2+}},$ which decrease as the exposure time passes under the direct action of accelerated electrons, is directly proportional to the flux density *φ_N_*, the interaction cross section σ, and the number of Fe^2+^ ions present in the beef samples at a given time. The reduction of $N_{{\mathrm{Fe}}^{2+}}$ caused by the direct action of electrons during the exposure time $t_{\exp}$ can be expressed as:

(3) $$\frac{dN_{{\mathrm{Fe}}^{2+}}}{dt_{\exp}}=-\varphi_{N}\sigma N_{{\mathrm{Fe}}^{2+}},$$

Since the sum of the HbO_2_ and metHb molecules remains constant over the observation time, and metHb molecules are absent in the beef samples at the start of irradiation, as shown in Formula (3), an increase in the number of metHb molecules in the beef samples during the irradiation can be expressed as:

(4) $$N_{1}\left(t_{\exp}\right)=N_{{\mathrm{Fe}}^{2+}}-N_{{\mathrm{Fe}}^{2+}}e^{-\varphi_{N}\sigma t_{\exp}}=N_{{\mathrm{Fe}}^{2+}}\left(1-e^{-\varphi_{N}\sigma t_{\exp}}\right),$$

Considering that the dose absorbed by the beef samples is proportional to the irradiation time and the dose rate *P*, which is equal to 5.0 ± 0.6 Gy/s, the dependency of metHb molecules that are contained in ensemble *N*_1_ on the irradiation dose is described by:

(5) $$N_{1}\left(D\right)=N_{{\mathrm{Fe}}^{3+}}\left(t_{\exp}\right)=N_{{\mathrm{Fe}}^{2+}}\left(1-e^{-\varphi_{N}\sigma PD}\right)=N_{{\mathrm{Fe}}^{2+}}\left(1-e^{-\alpha D}\right),$$

where α is a proportionality factor equal to 10^−3^ Gy^−1^.

Figure 5b shows the experimental dependency of *C*_metHb_ in the beef samples on the irradiation dose and the approximation function calculated using Formula (5), which is expressed as:

(6) $$N_{1}\left(D\right)=50\%\left(1-e^{-0.0014D}\right),$$

where $N_{{\mathrm{Fe}}^{2+}}$ is equal to 50 ± 1%, and α is equal to 0.0014 ± 0.0001 Gy^−1^. As can be seen, Formula (6) adequately describes the experimental data, since the standard approximation error is only marginal.

Figure 5b implies that only half of the $N_{{\mathrm{Fe}}^{2+}}$ ions in the beef samples irradiated with 1 MeV electrons in the dose of 10,000 Gy interacted with the electrons, which can be proved by the following factors determining how accelerated electrons interact with the molecules and atoms of biological objects:

- Nonuniform depth distribution of electron energy losses $\left(\frac{dE}{dx}\right)$ in the beef sample;
- A decline in the electron beam fluence as electrons penetrate deeper layers of the beef samples;
- Nonuniform depth–dose distribution in the beef samples;
- Discrete character of the interaction of electrons with the matter.

**Figure 6:**
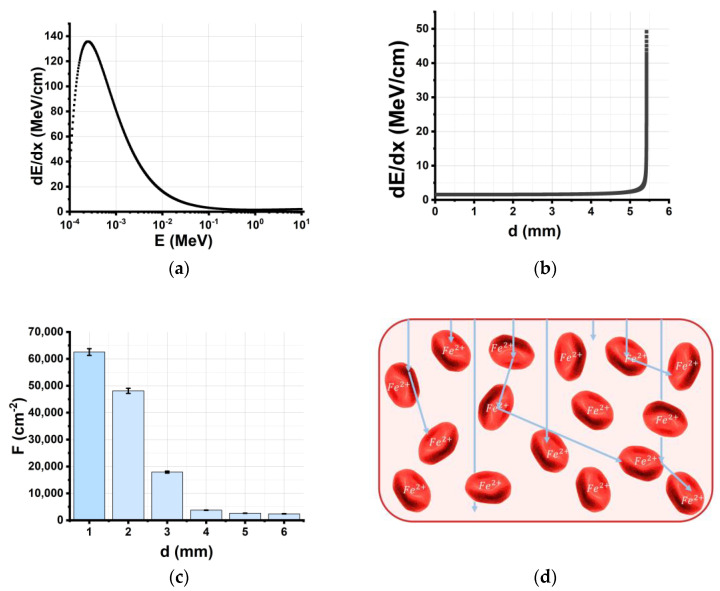
(**a**) Simulated dependency of electron energy losses $\left(\frac{dE}{dx}\right)$ (MeV/cm) in the water on the kinetic energy of electrons *E* (MeV); (**b**) simulated depth distribution of 1 MeV electron energy losses $\left(\frac{dE}{dx}\right)$ (MeV/cm) in the water; (**c**) simulated depth distribution of electron beam fluence *F* (cm^−2^) in the water; (**d**) random scattering and absorption of electrons in the beef samples.

Figure 6a shows the dependency of electron energy losses $\left(\frac{dE}{dx}\right)$ in the water on the kinetic energy of electrons estimated using the Geant4 tool kit. It suggests that the electron energy decreases as a result of electron energy losses, which causes the electron beam energy spectrum to disperse. As the electron beam passes through the water, electrons lose more energy, and the highest energy loss of 50 MeV/cm occurs at the maximum penetration depth of 5.5 mm (Figure 6b). Figure 6d suggests that an increase in electron penetration depth of up to 6 mm causes a 100-fold decrease in the electron beam fluence *F*. With all the factors put together, the depth distribution of the absorbed dose has a local maximum at a depth of 1.5 mm when the 6 mm water layer is irradiated with the 1 MeV electron beam (Figure 2b), which justifies the need for bilateral irradiation of the 6 mm thick beef pieces.

Apart from the physics of the direct interaction of electrons with matter, the spatial distribution of hemoglobin molecules in the beef samples has an impact on the concentration of metHb molecules (Figure 6c). According to calculations, the concentration of $N_{{\mathrm{Fe}}^{2+}}$ ions in the 4 g beef samples is approximately 6·10^16^ cm^−3^; thus, *N*_Fe_^2+^ ≈ 10^10^ are evenly distributed in a 1 cm^2^ surface layer of the beef sample. Figure 6d suggests that, while in the surface layer of the 6 mm thick beef sample, one electron interacts with one iron ion, and at a depth of 4 mm, there are 100 iron ions per one electron. This nonuniformity of interactions is the reason why not all HbO_2_ molecules transform into metHb in the beef samples irradiated with 10,000 Gy—which is the highest dose applicable to food irradiation. Since the metHb level in the beef samples after irradiation can serve as an indicator of irradiation nonuniformity and the discrete nature of the interaction between electrons and biological objects, not all bacteria will be inhibited even if the highest dose of 10,000 Gy is used for the irradiation of biological items.

The distinct dose-response relationship between *C*_metHb_ and the irradiation dose allows us to determine the dose absorbed by biological objects after exposure to ionizing radiation. Thus, this result could be used as a basis for the development of biodosimetry in food irradiation.

#### 3.2.2. The Kinetics of Ensemble N_2_

Since ensemble *N*_2_ is determined purely by bacterial activity in the beef samples (Figure 5b), metHb molecules in ensemble *N*_2_ are proportional to the number of bacteria *x* at the time of observation. According to the Verhulst population growth model [45], the kinetics of bacteria population *x* is expressed as:

(7) $$x\left(t\right)=\frac{x_{0}\varepsilon e^{\varepsilon t}}{\delta x_{0}e^{\varepsilon t}-\delta x_{0}+\varepsilon \;},$$

where *ε* is the growth rate, which is the difference between the reproduction rate *γ* and death rate σ of bacteria, *δ* is the competition rate, and *x*_0_ is the initial concentration of bacteria.

Suppose that the natural death rate of irradiated bacteria *σ* increases with a higher irradiation dose, while the reproduction rate of *γ* and the bacteria growth rate ε *=* γ − σ decreases when bacteria are exposed to accelerated electrons. In addition, the initial concentration of bacteria *x*_0_*(D)* goes down with an increase in the irradiation dose because of direct exposure to irradiation. Then the bacteria population after irradiation with dose *D* can be expressed by the following formula:

(8) $$x_{D}\left(t\right)=\frac{x_{0}\left(D\right)\varepsilon \left(D\right)e^{\varepsilon \left(D\right)t}}{\delta \left(D\right)x_{0}\left(D\right)e^{\varepsilon \left(D\right)t}-\delta \left(D\right)x_{0}\left(D\right)+\varepsilon \left(D\right)\;},$$

where ε(D), δ(D), and $x_{0}\left(D\right)$ are the radiation dose-dependent coefficients of the Verhulst model.

Since bacteria produce metHb [46], it can be assumed that the number of metHb molecules in ensemble *N*_2_ is proportional to the concentration of bacteria in the beef samples, and it can be expressed as:

(9) $$N_{2}\left(t\right)=kx\left(t\right),$$

where *k* is the proportionality coefficient.

The kinetics of ensemble *N*_2_ in the control nonirradiated samples can be described by the formula:

(10) $$N_{2\mathrm{control}}\left(t\right)=\frac{kx_{0}\varepsilon e^{\varepsilon t}}{\delta x_{0}e^{\varepsilon t}-\delta x_{0}+\varepsilon \;},$$

Figure 6c shows the dependency of *C*_metHb_ in the nonirradiated beef samples on the storage time, *t*_storage_, calculated using Formula (10). Taking into account the correlation between the number of metHb molecules and the bacteria population in the irradiated beef samples (9), the dependency of *C*_metHb_ in the irradiated samples on the storage time *t*_storage_ can be described by the formula:

(11) $$N_{2D}\left(t\right)=\frac{kx_{0}\left(D\right)\varepsilon \left(D\right)e^{\varepsilon \left(D\right)t}}{\delta \left(D\right)x_{0}\left(D\right)e^{\varepsilon \left(D\right)t}-\delta \left(D\right)x_{0}\left(D\right)+\varepsilon \left(D\right)\;},$$

Figure 6c shows the dependencies of *C*_metHb_ in the beef samples irradiated with the doses *D*_1_ and *D*_2_ calculated using Formula (11), which align with the experimental results presented in [35] and attest to the relevance of the Verhulst model used to describe the kinetics of bacteria populations in irradiated samples.

#### 3.2.3. Kinetics of Ensemble N_3_

Ensemble *N*_3_ is determined by the action of ROS that occurs in the nonirradiated beef samples during storage and in the irradiated beef samples because of an indirect action of accelerated electrons in addition. The formation of metHb molecules in the beef samples can occur because of the oxidation of Fe^2+^ ions by interaction with ROS. 

The increase in the number of metHb molecules in ensemble *N*_3_ as time passes is exponential [47] and can be expressed as:

(12) $$\frac{dN_{3}}{dt}=gN_{3},$$

where g is the metHb formation rate measured in units per day. Since the number of ROS depends on the irradiation dose absorbed by the beef samples, so does the coefficient g. Considering that, initially in the control nonirradiated samples, the number of metHb molecules is approximately *N*_3_*(t =* 0*) = N*_30_ = 0%, the initial number of metHb molecules in irradiated beef samples *N*_30_, occurring as a result of the indirect impact of irradiation, increases the higher the dose. So, the dependency of *C*_metHb_ in ensemble *N*_3_ on the storage time can be described by the formula:

(13) $$N_{3}\left(t\right)=N_{30}\left(D\right)e^{g\left(D\right)t},$$

Figure 6d shows the dependency of *N*_3_ in the beef samples irradiated with doses of *D*_1_ and *D*_2_ on the storage time calculated using Formula (13), assuming that in nonirradiated samples the HbO_2_ concentration increases 3% every 24 h in the presence of oxygen. It is clear from Figure 6d that with a higher irradiation dose, the initial number of metHb molecules *N*_30_ and metHb formation rate increase in the irradiated beef samples.

#### 3.2.4. Autoreduction of Methemoglobin to Oxyhemoglobin

Since the current research has shown that one hour after exposure, *C*_metHb_ in the beef samples irradiated with 250 Gy and 500 Gy is significantly higher than one day after exposure, it can be assumed that this phenomenon is triggered by autoreduction of metHb to HbO_2_.

Suppose *b* is the rate of the autoreduction of metHb to HbO_2_ in ensemble *N_4_*. It can be assumed that the amount of reduced metHb is proportional to its concentration at a given time. The reduction of metHb molecules in ensembles *N*_1_, *N*_2_, and *N*_3_ after irradiation over time can be expressed as follows:

(14) $$\frac{dN_{1}}{dt}=-bN_{1},$$

(15) $$\frac{dN_{2}}{dt}=-bN_{2},$$

(16) $$\frac{dN_{3}}{dt}=-bN_{3,}$$

where *b* is measured in units per day.

Taking into account Formulas (14)–(16), the change in the number of metHb molecules in ensembles *N*_1_, *N*_2_, and *N*_3_ over time because of autoreduction ($N_{1,2,3}^{red})$ can be expressed as follows:

(17) $$N_{1}^{\mathrm{red}}\left(t\right)=N_{1}\left(D\right)e^{-bt}=N_{{\mathrm{Fe}}^{2+}}\left(1-e^{-\alpha D}\right)e^{-bt},$$

(18) $$N_{2}^{\mathrm{red}}\left(t\right)=\frac{kx_{0}\left(D\right)\varepsilon \left(D\right)e^{\varepsilon \left(D\right)t}}{\delta \left(D\right)x_{0}\left(D\right)e^{\varepsilon \left(D\right)t}-\delta \left(D\right)x_{0}\left(D\right)+\varepsilon \left(D\right)\;}e^{-bt},$$

(19) $$N_{3}^{\mathrm{red}}\left(t\right)=N_{30}\left(D\right)e^{g\left(D\right)t}e^{-bt},$$

Figure 5e–g show the dependencies of *C*_metHb_ in ensembles *N*_1_, *N*_2_, and *N*_3_ in the beef samples irradiated with doses *D*_1_ and *D*_2_ on the storage time with and without autoreduction of metHb to HbO_2_. The given dependencies are calculated using Formulas (17)–(19).

To describe the kinetics of the methemoglobin concentration in the irradiated beef samples during storage, it is important to note that, on the one hand, there is an increase in the concentration of methemoglobin as a result of the direct action of accelerated electrons, bacterial activity, and oxidation because of ROS, and on the other hand, there is a decrease in its concentration because of autoreduction. The prevailing processes in the beef samples change over time to impact the methemoglobin concentration. While during irradiation, hemoglobin oxidation, as a result of the direct ionization of Fe^2+^ to Fe^3+^ and the action of ROS because of indirect action of accelerated electrons, plays the most important role, on day one of storage, the autoreduction of methemoglobin is the most prominent phenomenon occurring in the beef samples irradiated with relatively small doses. In contrast, the observation during day 2 and day 3 shows that bacterial activity, as well as ROS, which is formed because of lipid peroxidation and protein oxidation, come into the picture to accelerate hemoglobin oxidation. Moreover, all these processes, occurring at the same time, act upon each other during the entire period of storage. All these mechanisms are used as the basis for the mathematical model that we apply to describe the change in methemoglobin concentration in the irradiated and nonirradiated beef samples during storage.

In this model, hemoglobin derivatives are represented as independent ensembles *N*_1_, *N*_2_, *N*_3_, and *N*_4_ occurring for the different specific reasons described above. The total methemoglobin concentration in the beef samples is a superposition of all three ensembles, *N*_total_ = *N*_1_ + *N*_2_ + *N*_3_, while ensemble *N*_4_ contributes to each *N*_1_, *N*_2_, and *N*_3_ ensemble, making the amount of molecules in each of them grow. At the same time, the amount of molecules in each of the ensembles, *N*_1_, *N*_2_, and *N*_3_, decreases, prompting the molecules to transfer to ensemble *N_4_* because of autoreduction.

**Figure 7:**
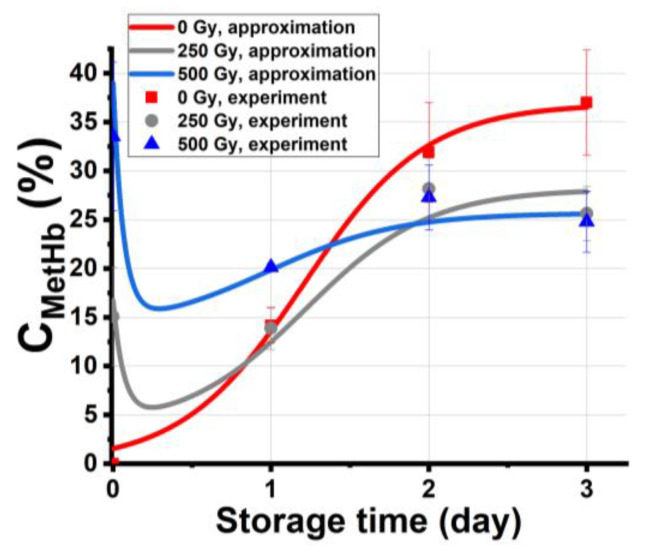
Experimental dependencies of *C*_metHb_ in the nonirradiated samples and the samples irradiated with 250 Gy and 500 Gy on the storage time *t*_storage_ and the approximation of experimental dependencies: nonirradiated samples: *x*_0_ = 1%, ε−b = 3.02 un. per day, *δ* = 0.08 un. per day, *N*_30_ = 1%, g = 0.03 un. per day, *R*^2^ = 0.98; 250 Gy: *N*_1_ = 15%, *x*_0_ = 1%, *ε* = 2.9 un. per day, *δ* = 0.13 un. per day, *b* = 16 Gy^−1^, *N*_30_ = 3.6%; g = 0.03 un. per day, *R*^2^ = 0.96; 500 Gy: *N*_1_ = 34%, *x*_0_ = 1%, *ε* = 2.8 un. per day, *δ* = 0.2 un. per day, *b* = 16 Gy^−1^, *N*_30_ = 11.7%, g = 0.03 un. per day. *R*^2^ = 0.97.

Figure 7 shows the experimental dependencies of *C*_metHb_ in the nonirradiated samples and the samples irradiated with 250 Gy and 500 Gy on the storage time, *t*_storage_. The graph also shows the approximation of experimental data calculated using Formulas (17)–(19), assuming that the total concentration of methemoglobin *N*_total_ in the beef samples is determined by all four ensembles swapping molecules during the entire period of storage.

The high values of correlation coefficients *R* = 0.96–0.98 attest to the adequacy of the model, which describes the change of methemoglobin concentration in the beef samples during storage after exposure to accelerated electrons. The proposed model describing the experimental results confirms that the dependencies of methemoglobin concentrations on the storage time and on the irradiation dose are essentially nonlinear, which corresponds to the experimental data obtained in the study.

## 4. Materials and Methods

### 4.1. Object of Study

Chilled tenderloin beef samples weighing (3.5 ± 0.5) g stored at 4 °C for one day after slaughter were used in the study to measure the hemoglobin concentration in irradiated and nonirradiated beef. For the purpose of the study, the beef tenderloin was cut into 48 equally sized parallelepipeds measuring 20 mm × 20 mm × 6 mm, which were placed in a ∅ 35 mm Petri dish for irradiation (Figure 1).

### 4.2. Electron Beam Irradiation

The beef samples were irradiated using a 1 MeV continuous electron accelerator UELR-1-25-T-001 (Skobeltsyn Scientific Research Institute of Nuclear Physics in cooperation with LLC NPP Torii, Moscow, Russia) with an average beam power of 25 kW.

Dishes with eight beef pieces in them were placed on a 35 cm × 5.2 cm duralumin plate located 12 cm from the beam output. Since the penetration depth of 1 MeV electrons in water does not exceed 5 mm, the samples were irradiated from two opposite sides to achieve uniform irradiation. A total of 10 irradiation sessions were performed for 5 doses at the rate of 2 irradiation sessions per dose (Figure 1).

During irradiation, charge *Q*_exp_, absorbed by the part of the duralumin plate not occupied by the samples, was recorded to determine the dose absorbed by the samples using ADC (OOO Oven, Moscow, Russia), and the margin of error in determining the charge was no more than 2%.

Table 1 presents the data on the average exposure time *t*_exp_ measured for each irradiation session and the average charge *Q*_exp_ absorbed by the duralumin plate.

The ambient temperature during irradiation was 20 °C, and the control samples were stored in the same temperature conditions as the samples exposed to electrons.

### 4.3. Computer Simulation to Determine the Dose Absorbed by Beef Samples

The dose absorbed by the beef samples and dose uniformity were determined using a Geant 4 tool kit (CERN, European Organization for Nuclear Research, Geneva, Switzerland).

During computer simulation, eight beef samples represented by 20 mm × 20 mm × 6 mm water parallelepipeds were irradiated with 10^10^ electrons having the energy spectrum shown in Figure 2a. Water was selected as a medium for the simulation since it is close in its properties to the range of biological objects involved in the study. The source of electrons was represented by a 100 mm × 100 mm square placed 12 cm from the duralumin plate on which the water parallelepipeds were located. During simulation, water phantoms representing beef samples were irradiated as described in Section 3.2 above.

The depth–dose distribution in the phantom was recorded using a 200 × 200 × 60 virtual grid dividing the phantom into 60 0.1 mm thick layers. To determine the dose distribution, the sum of the energies absorbed in cell ∑*E_i_*, the sum of the squares of the energies absorbed during individual interactions ∑*E^2^_i_*, and the number of interactions that occurred in cell *N*_i_, where *i* is the cell number, were recorded in each 1 mm × 1 mm × 1 mm cell. The total charge absorbed by the phantom when it was irradiated with 10^10^ electrons was also registered. The average value of the absorbed dose *D_i_* (20) and its standard deviation *S_i_* (21) in the *i* cell were calculated using the following formulas:

(20) $$D_{i}=\frac{\frac{1}{N_{i}}\sum_{j=1}^{N_{i}}E_{ij}}{m_{i}},$$

(21) $$S_{i}=\sqrt{\left(\frac{1}{N_{i}-1}\frac{1}{m_{i}^{2}}\sum_{j=1}^{N_{i}}E_{ij}^{2}-{\left(\frac{1}{N_{i}-1}\frac{1}{m_{i}}\sum_{j=1}^{N_{i}}E_{ij}\right)}^{2}\right)},$$

where $m_{i}$ is the mass of the *i* cell. The total charge *Q*_mod_ absorbed by the phantom when irradiated with 10^10^ electrons was also estimated.

The dose absorbed by the phantom during simulation was calculated using the following formula:

(22) $$D_{\mathrm{mod}}=\frac{\sum_{i=1}^{N}D_{i}}{m},$$

where *m* is the mass of the phantom, and *N* is the total number of cells. The error in determining the dose absorbed by the phantoms during the simulation did not exceed 2%.

To determine the dose absorbed by the beef samples *D*_exp_ during irradiation, the charge absorbed by the samples *Q* was calculated, taking into account the charge absorbed by the duralumin plate *Q*_exp_, using the following formula:

(23) $$Q=\frac{S_{\mathrm{sample}}}{S_{\mathrm{dur}}-nS_{\mathrm{sample}}\;}\cdot Q_{\exp}=\frac{S_{\mathrm{sample}}}{S_{\mathrm{dur}}-nS_{\mathrm{sample}}}\cdot \frac{I\Delta t_{\exp}}{\bar{e}},$$

where *S*_sample_ = 9.6 cm^2^ is the area occupied by the beef samples placed in a ∅ 35 mm Petri dish, which was placed on the duralumin plate, *S*_dur_ = 182 cm^2^ is the area of the duralumin plate, *n* = 8 is the number of the beef samples on the plate during irradiation, *I* is the average value of beam current, *t*_exp_ is the time of exposure, and $\bar{e}$ is the electron charge. The doses absorbed by the samples that are proportional to the total electron charge *Q* can be calculated using the following formula:

(24) $$D_{\exp}=\frac{D_{\mathrm{mod}}}{Q_{\mathrm{mod}}\;}\cdot Q,$$

Time of exposure *t*_exp_ (s) during each irradiation session and charge *Q*_exp_ (nC) absorbed by duralumin plate, as well as doses *D*_exp_ (Gy) absorbed by the samples, are shown in Table 1. Table 1 represents the doses *D*_exp_ (Gy) calculated using Formula (24) and the rounded values of doses *D*_exp_, which were used to construct experimental and analytical dependencies.

### 4.4. Spectrophotometry of Hemoglobin Derivatives

To obtain the absorption spectrum of the hemoglobin derivatives, each beef sample was placed in a 5 mL 0.01M phosphate buffer solution with a NaCl concentration of 0.137 mol/L. After 15 min, 1.5 mL of supernatant solution was poured into 2 mL Eppendorf tubes and then centrifuged using a Universal 320 centrifuge (Andreas Hettich GmbH & Co. KG, Tuttlingen, Germany) at 3500 rpm for 5 min. The experimental spectra of the solutions *A*^exp^(*λ*) were measured at wavelength ranging from 190 nm to 1100 nm in 2 nm increments using a Unico 2800 spectrophotometer (United Products & Instruments, Dayton, NJ, USA).

### 4.5. Hemoglobin Derivative Concentration

Nonlinear curve fitting of the experimental spectra of solutions *A*(*λ_l_*) using an Origin Pro 2019 (OriginLab Corporation, Northampton, MA, USA) was performed to determine the hemoglobin derivative concentration in the beef samples. The fitting function corresponding to the hemoglobin absorption law was represented using the following formula:

(25) $$A_{l}{\left(\lambda_{l}\right)}_{\mathrm{theory}}=\varepsilon_{\mathrm{Hb},l}C_{\mathrm{Hb}}L+\varepsilon_{{\mathrm{HbO}}_{2},l}C_{{\mathrm{HbO}}_{2}}L+\varepsilon_{\mathrm{MetHb},l}C_{\mathrm{MetHb}}L+\frac{E}{\lambda^{4}}+K,$$

where *l* is the number of wavelengths; ε_Hb,l_(*λ_l_*), ε_HbO_2_,l_(*λ_l_*), and ε_MetHb,l_(*λ_l_*) are molar absorption coefficients of deoxyhemoglobin, oxyhemoglobin, and methemoglobin, respectively; *C*_Hb_, *C*_HbO_2__, and *C*_MetHb_ are the concentrations of deoxyhemoglobin, oxyhemoglobin, and methemoglobin, respectively; *L* = 1 cm is the thickness of the solution layer; *E* and *K* are scattering coefficients [25,40]. According to the current research literature, the molar absorption coefficient of beef erythrocyte hemoglobin derivatives practically coincides with those of human hemoglobin [48]. The concentrations of deoxyhemoglobin, oxyhemoglobin, and methemoglobin, as well as scattering coefficients *E* and *K*, were calculated using the Levenberg–Marquardt algorithm. Next, the dependencies of the methemoglobin concentration *C*_metHb_ in the beef samples irradiated with different doses on the storage time, *t*_storage_, as well as the dependency of *C*_metHb_ on the dose absorbed by the beef samples, were plotted to estimate the impact of accelerated electron irradiation on hemoglobin derivatives in beef.

## 5. Conclusions

This experimental study, which involves the development of an express spectrophotometry method estimating the oxidation level in biological objects containing red blood cells, shows the nonlinear dependencies of methemoglobin concentration in beef irradiated with accelerated electrons on both storage time and irradiation dose during the three days after exposure. Interestingly, three days after irradiation, the concentration of methemoglobin in the beef irradiated with doses of up to 1000 Gy is lower compared with the nonirradiated beef samples, while the doses exceeding 1000 Gy increase methemoglobin concentration in the beef. It can be noted that the methemoglobin rate in beef after exposure to accelerated electrons is a potential quantitative marker of the oxidation level for foods containing red blood cells, as well as the bacterial content inhibited by irradiation. These findings can be used to determine the optimal irradiation parameters specifically for beef irradiation guidelines. The distinct dose-response dependency of the methemoglobin rate in beef irradiated with doses ranging from 100 Gy to 5000 Gy can be used as a basis for the development of an express biodosimetry method in food irradiation for products containing red blood cells.

The proposed model describes the change in hemoglobin derivative concentration in beef after electron beam irradiation taking into account three oxidation mechanisms—direct ionization caused by accelerated electrons, biophysical and biochemical processes as a result of bacterial activity, and oxidation because of reactive oxygen species. This model can be the basis for theoretical and practical solutions in the field of food irradiation.

In addition, the methodology presented in this study will be useful for research into the radiobiology and biophysics of radiation effects.

## Figures and Tables

**Table 1:** Irradiation parameters for the electron processing of beef samples.

Irradiation Session	*D*_exp_ (Gy)	*t*_exp_ (s)	*Q*_exp_ (nC)	*D* (Gy)
1, 2	248 ± 6	55 ± 1	16,310 ± 285	250
3, 4	502 ± 10	100 ± 1	32,700 ± 423	500
5, 6	1018 ± 20	186 ± 1	65,058 ± 1112	1000
7, 8	5023 ± 50	988 ± 1	325,165 ± 5870	5000
9, 10	10,037 ± 90	1864 ± 1	650,108 ± 8860	10,000

## Data Availability

Not applicable.

## Sample Availability

Tenderloin beef samples used in the study are readily available on food markets.
